# FAIR principles for AI models with a practical application for accelerated high energy diffraction microscopy

**DOI:** 10.1038/s41597-022-01712-9

**Published:** 2022-11-10

**Authors:** Nikil Ravi, Pranshu Chaturvedi, E. A. Huerta, Zhengchun Liu, Ryan Chard, Aristana Scourtas, K. J. Schmidt, Kyle Chard, Ben Blaiszik, Ian Foster

**Affiliations:** 1 Data Science and Learning Division, Argonne National Laboratory, Lemont, Illinois 60439 USA; 2 Department of Computer Science, University of Illinois at Urbana-Champaign, Urbana, Illinois 61801 USA; 3 Department of Mathematics, University of Illinois at Urbana-Champaign, Urbana, Illinois 61801 USA; 4 Department of Computer Science, University of Chicago, Chicago, Illinois 60637 USA; 5 Globus, University of Chicago, Chicago, Illinois 60637 USA

**Keywords:** Policy, Research data

## Abstract

A concise and measurable set of FAIR (Findable, Accessible, Interoperable and Reusable) principles for scientific data is transforming the state-of-practice for data management and stewardship, supporting and enabling discovery and innovation. Learning from this initiative, and acknowledging the impact of artificial intelligence (AI) in the practice of science and engineering, we introduce a set of practical, concise, and measurable FAIR principles for AI models. We showcase how to create and share FAIR data and AI models within a unified computational framework combining the following elements: the Advanced Photon Source at Argonne National Laboratory, the Materials Data Facility, the Data and Learning Hub for Science, and funcX, and the Argonne Leadership Computing Facility (ALCF), in particular the ThetaGPU supercomputer and the SambaNova DataScale^®^ system at the ALCF AI Testbed. We describe how this domain-agnostic computational framework may be harnessed to enable autonomous AI-driven discovery.

## Introduction

Innovation at the interface of artificial intelligence (AI) and high performance computing is powering breakthroughs in science, engineering, and industry^1–11^. Thus, it is timely and important to define best AI practices that facilitate cross-pollination of expertise, reduce time-to-insight, increase reusability of scientific data and AI models by humans and machines, and reduce duplication of effort. To realize these goals, researchers are working to understand how to adapt FAIR guiding principles^12,13^–originally developed in the context of digital assets, such as data and the tools, algorithms, and workflows that produce such data–to streamline the development and adoption of AI methodologies.

FAIR guiding principles in the context of scientific datasets aim to accelerate innovation and scientific discovery by defining and implementing best practices for data stewardship and governance that enable the automation of data management. FAIR scientific datasets are also AI-ready when they are shared and published in suitable formats (HDF5^14^ or ROOT^15^) that facilitate their use in modern computing environments and with open source APIs for AI research (TensorFLow^16^ or PyTorch^17^).

The creation of FAIR and AI-ready datasets is transforming the state-of-practice of AI research across disciplines^9,18–20^. In view of these activities, and given the growth and impact of AI for Science programs, it is critical to define at a practical level what FAIR means for AI models–the theme of this article. To contextualize the FAIR principles for AI models that we introduce in this article, we begin by describing a FAIR and AI-ready scientific dataset that we used to create and publish FAIR AI models. We do this because there is an agreed-upon set of guidelines to FAIRify scientific datasets^9,12,13^ that we take as the basis, or common ground, from which we define a set of practical FAIR principles for AI models.

To quantify the FAIRness of our AI models, we have created a domain-agnostic computational framework that brings together advanced scientific data infrastructure and modern computing environments to conduct automated, accelerated, and reproducible AI inference. This computational framework facilitates the integration of FAIR and AI-ready datasets with FAIR AI models. This unified approach will enable researchers to obtain a clear understanding of the state-of-practice of AI to address contemporary scientific grand challenges. Understanding needs and gaps in available datasets and AI models will catalyze the sharing of knowledge and expertise at an accelerated pace and scale, thereby enabling focused R&D in areas where AI capabilities are currently lacking. The components of this computational framework encompass:

- Data and Learning Hub for Science (https://www.dlhub.org, DLHub)^21,22^ to publish and share FAIR AI models,
- Materials Data Facility (https://materialsdatafacility.org, MDF)^23^ to publish and share FAIR and AI-ready datasets,
- funcX, https://funcx.org^24^, a distributed Function as a Service (FaaS) platform, to connect FAIR AI models hosted at DLHub and FAIR and AI-ready datasets hosted at MDF with the ThetaGPU supercomputer at the Argonne Leadership Computing Facility (https://www.alcf.anl.gov, ALCF) to conduct reproducible AI-driven inference.

Below we describe how to use this domain-agnostic computational framework to evaluate the FAIRness of AI models. We also describe how to integrate uncertainty quantification metrics in AI models to assess the reliability of their predictions. Throughout our discussion, we leverage disparate hardware architectures, ranging from GPUs to the SambaNova Reconfigurable Dataflow UnitTM (RDU) at the ALCF AI-Testbed, https://www.alcf.anl.gov/alcf-ai-testbed, to ensure that our ideas provide useful, easy-to-follow guidance to a diverse ecosystem of researchers and AI practitioners.

**Fig. 1:**
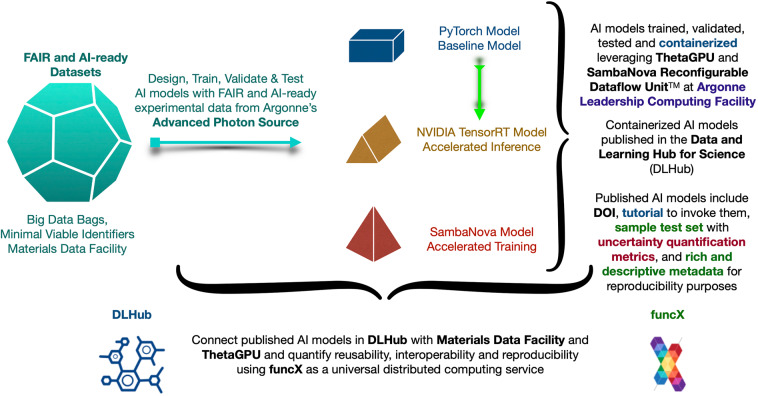
FAIR AI models and data. Proposed approach to combining FAIR and AI-ready experimental data, FAIR AI models and scientific data, and computing infrastructure to accelerate and automate scientific discovery.

We release our FAIR AI datasets and models, as well as scientific software to enable other researchers to reproduce our work, and to engage in meaningful interactions that contribute towards an agreed upon, community wide definition of what FAIR means in the context of AI models, with an emphasis on practical applications. Figure 1 summarizes our proposed approach to creating FAIR AI models and experimental datasets. This approach has three main pillars, namely: (i) the creation and sharing and FAIR and AI-ready datasets; (ii) the combination of such datasets with modern computing environments to streamline and automate the creation and publication of FAIR AI models; and (iii) the combination of FAIR datasets and AI models with scientific data infrastructure and advanced computing resources to automate accelerated AI inference with well-defined uncertainty quantification metrics to measure the reliability, reproducibility, and statistical robustness of AI predictions.

We selected high energy diffraction microscopy as a science driver for this work^25^. This technique is used to characterize 3D information about the structure of polycrystalline materials through the identification of Bragg diffraction peaks. The data used for the identification of Bragg peaks with our AI models was produced at the Advanced Photon Source, https://www.aps.anl.gov, at Argonne National Laboratory. While we guide the discussion of our methods with this application, the definitions, approaches and computational framework introduced in this article are domain-agnostic and may be harnessed for any other scientific application.

## Results

We present two main results:

- **FAIR and AI-ready experimental datasets**. We published experimental datasets used to train, validate and test our AI models via the MDF, and created Big Data Bags (BDBags)^26^ with associated Minimal Viable Identifiers (minids)^26^ to specify, describe, and reference each of these datasets. We also published Jupyter notebooks via MDF that describe these datasets and illustrate how to explore and use them to train, validate, and test AI models. We FAIRified these datasets following practical guidelines^9,12,13^.
- **Definition of FAIR for AI models and practical examples**. We use the aforementioned FAIR and AI-ready datasets to produce three AI models: a baseline PyTorch model, an optimized AI model for accelerated inference that is constructed by porting the baseline PyTorch model into an NVIDIA TensorRT engine, and a model created on the SambaNova DataScale^®^ system at the ALCF AI-Testbed. We use the ThetaGPU supercomputer at ALCF to create and containerize the first two models. We use these three models to showcase how our proposed definitions for FAIR AI models may be quantified by creating a framework that brings together DLHub, funcX, the MDF and disparate hardware architectures at ALCF.

### FAIR and AI-ready datasets

We FAIRified a high energy microscopy dataset produced at the Advanced Photon Source at Argonne National Laboratory. We split this dataset into a training dataset that we used to create the AI models described below, and a validation dataset that we used to compute relevant metrics pertaining to the performance of our AI models for regression analyses. We published these datasets via MDF and provide the following information:

**Findable**

- Unique Digital Object Identifier (DOI) for Training_Set^27^ andValidation_Set^28^
- Rich and descriptive metadata
- Detailed description of the datasets, including data type and shape
- Metadata uses machine-readable keywords
- Metadata contains resource identifier
- (Meta)data are indexed in a searchable resource

**Accessible**

- Datasets are published with CC-BY 4.0 licenses
- Training_Set^27^ and Validation_Set^28^ are open datasets
- (Meta)data are retrievable by their identifier using a standardized communications protocol
- Metadata remains discoverable, even in the absence of the datasets

**Interoperable**

- Datasets are published in open HDF5 format
- Metadata contains qualified references to related data^29^ and publications^25^
- Metadata follows standards for X-ray spectroscopy, uses controlled vocabularies, ontologies and good data model following a physics classification scheme developed by the American Physical Society (APS)^30^. These datasets were collected and curated to streamline their use for AI analyses^31^
- Uses FAIR vocabulary following the ten rules provided in ref. ^32^

**Reusable**

- Author list, and points of contact including email addresses
- Datasets include Jupyter notebooks to explore, understand and visualize the datasets
- Datasets include Jupyter notebooks that show how to use the Training_Set^27^ to train AI models with the scripts we have released in GitHub^33^
- Datasets include Jupyter notebooks that show how to use the Validation_Set^28^ to conduct AI inference using the computational framework we described above
- Datasets include a description of how they were produced and provide this information in a machine-readable metadata format

We have also created BDBags and minids for each of these datasets to ensure that creation, assembly, consumption, identification, and exchange of these datasets can be easily integrated into a user’s workflow. A BDBag is a mechanism for defining a dataset and its contents by enumerating its elements, regardless of their location. Each BDBag has a data/directory containing (meta)data files, along with a checksum for each file. A minid for each of these BDBags provides a lightweight persistent identifier for unambiguously identifying the dataset regardless of their location. Computing a checksum of BDBag contents allows others to validate that they have the correct dataset, and that there is no loss of data. In short, these tools enable us to package and describe our datasets in a common manner, and to refer unambiguously to the datasets, thereby enabling efficient management and exchange of data.

1. BDBag for training set^34^ and its associated minid:olgmRyIu8Am7
2. BDBag for validation set^35^ with its associated minid:16RmizZ1miAau

In addition to the FAIR properties listed above, we have ensured that our datasets meet the detailed FAIR guidelines described in ref. ^9^.

### FAIR AI models

The understanding of FAIR principles in the creation and sharing of AI models is an active area of research. Here we contribute to this effort by introducing a set of measurable FAIR principles for AI models, with an emphasis on practical applications. We propose that all these principles are quantified as Pass or Fail. We have constructed the definitions of FAIR principles for AI models taking into account the diverse needs and level of expertise of AI practitioners. In practice, we have considered three types of AI models, which have the same AI architecture, as described in this GitHub repository^33^, and which are trained with the same Training_Set^27^. However, as shown in Fig. 1 these AI models have distinct features: (i) a baseline AI model was trained using PyTorch on GPUs in the ThetaGPU supercomputer at ALCF; (ii) the fully trained AI model in (i) was optimized with NVIDIA TensorRT, a software development kit (SDK) that enables low latency and high throughput AI-inference, in the ThetaGPU supercomputer at ALCF; and (iii) the AI model in (i) was trained using the SambaNova DataScale^®^ system at the ALCF AI-Testbed. These cases exemplify different needs and levels of expertise in the development of AI tools. However, we show below that irrespective of the skill set of AI practitioners, hardware used, and target application, all these fully trained AI models deliver consistent results. In what follows, an AI model refers to a fully trained AI model.

#### Findable

##### Proposition

An AI model is findable when a DOI may direct a human or machine to a digital resource that contains all the required information to define uniquely the AI model, i.e., descriptive and rich **AI model metadata** that provides the title of the model, authors, DOI, year of publication, free text description, information about the input and output data type and shape, dependencies (TensorFlow, PyTorch, Conda, etc.) and their versions used to create and containerize the AI model. The published AI model should also include instructions to run it, a minimal test set to evaluate its performance, and the actual fully trained AI model in a user-/machine-friendly format, such as a Jupyter notebook or a container.

This work We have published three AI models in DLHub, and assigned DOIs to each of them: (i) a traditional PyTorch model; (ii) an NVIDIA TensorRT version of the traditional PyTorch model; and (iii) a model trained on the SambaNova DataScale^®^ system:

1. PyTorch Model^36^
2. TensorRT Model^37^
3. SambaNova Model^38^

Each of these AI models includes rich and descriptive metadata following the DataCite metadata standard, and is available as JSON formatted responses through a REST API or Python SDK. Furthermore, DLHub provides an interface that enables users to seamlessly run AI models following step-by-step examples, and explore the AI model’s metadata in detail.

#### Accessible

##### Proposition

An AI model is accessible when it is discoverable by a human or machine, and it may be downloaded (to further develop it, retrain it, optimize it for accelerated inference, etc.) or directly invoked to conduct AI inference. The AI model’s metadata should also be discoverable even in the absence of the AI model.

This work Our AI models are freely accessible through their DOIs provided by DLHub. The AI models may be freely downloaded or invoked over the network for AI inference. To do the latter, we have deployed funcX endpoints at the ThetaGPU supercomputer, which may be used to invoke the AI models and access the datasets that we have published at DLHub and the MDF, respectively. Users may interact with published AI models in DLHub by submitting HTTP requests to a REST API. In essence, the DLHub SDK contains a client that provides a Python API to these requests and hides the tedious operations involved in making an HTTP call from Python. The schema used by DLHub is such that the AI model’s metadata provides explicit information about the AI model’s identifier and its type (in this case a DOI). Thus, this schema ensures that the AI model’s metadata remains discoverable, even in the absence of the AI model.

#### Interoperable

##### Proposition

An AI model is interoperable when it may be readily used by machines to conduct AI-driven inference across disparate hardware architectures. This property may be realized by containerizing the model and providing infrastructure to enable AI models to process data in disparate hardware architectures. Furthermore, the AI model’s metadata should use a formal, accessible, and broadly used format, such as JSON or HTML.

This work We have quantified this property by evaluating the performance of our AI models across disparate hardware architectures. For instance, we have run our three AI models using RDUs, CPUs, and GPUs available at ALCF. Furthermore, as we describe below, we published these models in DLHub with metadata available as both JSON and HTML. Furthermore, the AI models’ metadata in DLHub follows the vocabulary of the DataCite metadata standard.

#### Reusable

##### Proposition

An AI model is reusable when it may be used by humans or machines to reproduce its putative capabilities for AI-driven analyses, and when it contains quantifiable metrics that inform users whether it may be used to process datasets that differ from those originally used to create it. This property can be achieved by providing information about the AI model’s required input and output data types and shapes; examples that show how to invoke the model; and a control dataset and uncertainty quantification metrics that indicate the realm of usability of the model. These reusability metrics may also be used to identify when a model is no longer trustworthy, in which case active learning, transfer learning, or related methods may be needed to fine tune the model so it may provide trustworthy predictions. The AI model’s metadata should also provide detailed provenance about the AI model, i.e., authors, dependencies and their versions used to create and containerize the model, datasets used to train the model, year of publication, and a brief description of the model.

This work Our models published in DLHub include examples that describe the input data type and shape, the output data type and shape, and a sample dataset to quantify their performance. We have also provided domain-informed metrics to ascertain when the predictions of our AI models are trustworthy. DLHub SDK provides step-by-step examples that show how to run the models and explore the AI model’s metadata, which provides detailed provenance of the AI model. In this study we use the L2 norm or Euclidean distance, a well-known metric for quantifying the performance of AI models for regression.

### Expected outcome of proposed FAIR principles for AI models

In the same vein as FAIR principles for scientific data aim to automate data management, the FAIR principles for AI models we proposed above aim to maximize the impact of AI tools and methodologies, and to facilitate their adoption and further development with a view to eventually realize autonomous AI-driven scientific discovery. To realize that goal it is essential to link FAIR and AI-ready datasets with FAIR AI models within a flexible and smart computing fabric that streamlines the creation and use of AI models in scientific discovery. The domain-agnostic computing framework we described above represents a step in that direction, since it brings together scientific data infrastructure (DLHub & funcX & Globus), modern computing environments (ALCF and ALCF AI-Testbed), and leverage FAIR & AI-ready datasets (MDF).

This ready-to-use framework addresses common challenges in the adoption and use of AI methodologies, namely, it provides examples that illustrate how to use AI models, and their expected input and output data; it enables users to readily use, download, or further develop AI models. We have created this framework because, as AI practitioners, we are acutely aware that a common roadblock to using existing AI models is that of deploying a model on a computing platform but finding that, for example, library incompatibilities slow down progress and lead to duplication of efforts, or discourage researchers from investing limited time and resources in the adoption of AI methodologies. Our proposed framework addresses these limitations, and demonstrates how to combine computing platforms, container technologies, and tools to accelerate AI inference. Our proposed framework also highlights the importance of including uncertainty quantification metrics in AI models that inform users on the reliability and realm of applicability of AI predictions.

## Methods

### Dataset description

The sample dataset we used to train and evaluate BraggNN^25^ was collected by using an undeformed bi-crystal Gold sample^39^ with 1440 frames (0.25° steps over 360°) totaling 69347 valid Bragg peaks. Each image is 11 × 11 pixels. The spatial resolution of the data is such that each pixel is 200 *μm*. We used 80% of these peaks (55478) as our training set, 6000 peaks (~9%) as our validation set for early stopping^40^, and the remaining 7869 peaks (~11%) as a test set. We also created a smaller validation dataset consisting of 13799 samples taken from the training dataset, which we used to compute and report relevant metrics.

### AI Models description

We present three AI models, namely, (i) a traditional PyTorch model; (ii) an NVIDIA TensorRT engine that optimizes our traditional PyTorch for accelerated inference; and (iii) a model trained with the SambaNova DataScale^®^ system at the ALCF AI-Testbed.

#### PyTorch model

Our base AI model is implemented with the PyTorch framework. We trained the model for 500 epochs with a mini-batch size of 512, and used validation-based early stopping to avoid overfitting. Training takes around two hours with an NVIDIA V100 GPU in the ThetaGPU supercomputer. As mentioned above, the input data are images 11 × 11 pixels. The AI model’s output is a 2D list of Bragg peak positions in terms of pixel locations.

#### Model Conversion to TensorRT

We use the Open Neural Network Exchange (ONNX) converter within PyTorch to convert our PyTorch BraggNN model into the ONNX format. We then use TensorRT by invoking a Singularity container^41^ that includes TensorRT,ONNX,PyTorch,PyCUDA and other common deep learning libraries to build a TensorRT engine and save it in a.plan format. We used the following parameters when building our engine: the maximum amount of memory that can be allocated by the engine, set to 32 GB, allowed the TensorRT graph optimizer to make opportunistic use of half-precision (FP16) computation when feasible; input dimensions, including the batch size, of (16384,1,11,11); output dimensions of (16384,1,11,11); and a flag that serializes the built engine so that the engine will not have to be reinitialized in subsequent runs. TensorRT applies a series of optimizations to the model by running a GPU profiler to find the best GPU kernels to use for various neural network computations, applying graph optimization techniques to reduce the number of nodes and edges in a model such as layer fusion, and quantization where appropriate. Finally, we create an inference script using PyCUDA that allocates memory for the model and data on the GPU and makes the appropriate memory copies between the CPU and GPU in order to perform accelerated inference with our TensorRT engine.

#### SambaNova model

The SambaNova DataScale^®^ system at the ALCF AI-Testbed uses SambaFlow^TM^ software, which has been integrated with popular open source APIs, such as PyTorch and TensorFlow. Leveraging these tools, we used SambaFlow to automatically extract, optimize, and execute our originally PyTorch BraggNN model with SambaNova’s RDUs^42^. We find that the predictions of our SambaNova BraggNN model are consistent with those obtained with PyTorch and TensorRT models.

#### Benchmark results in the ThetaGPU supercomputer

**Fig. 2:**
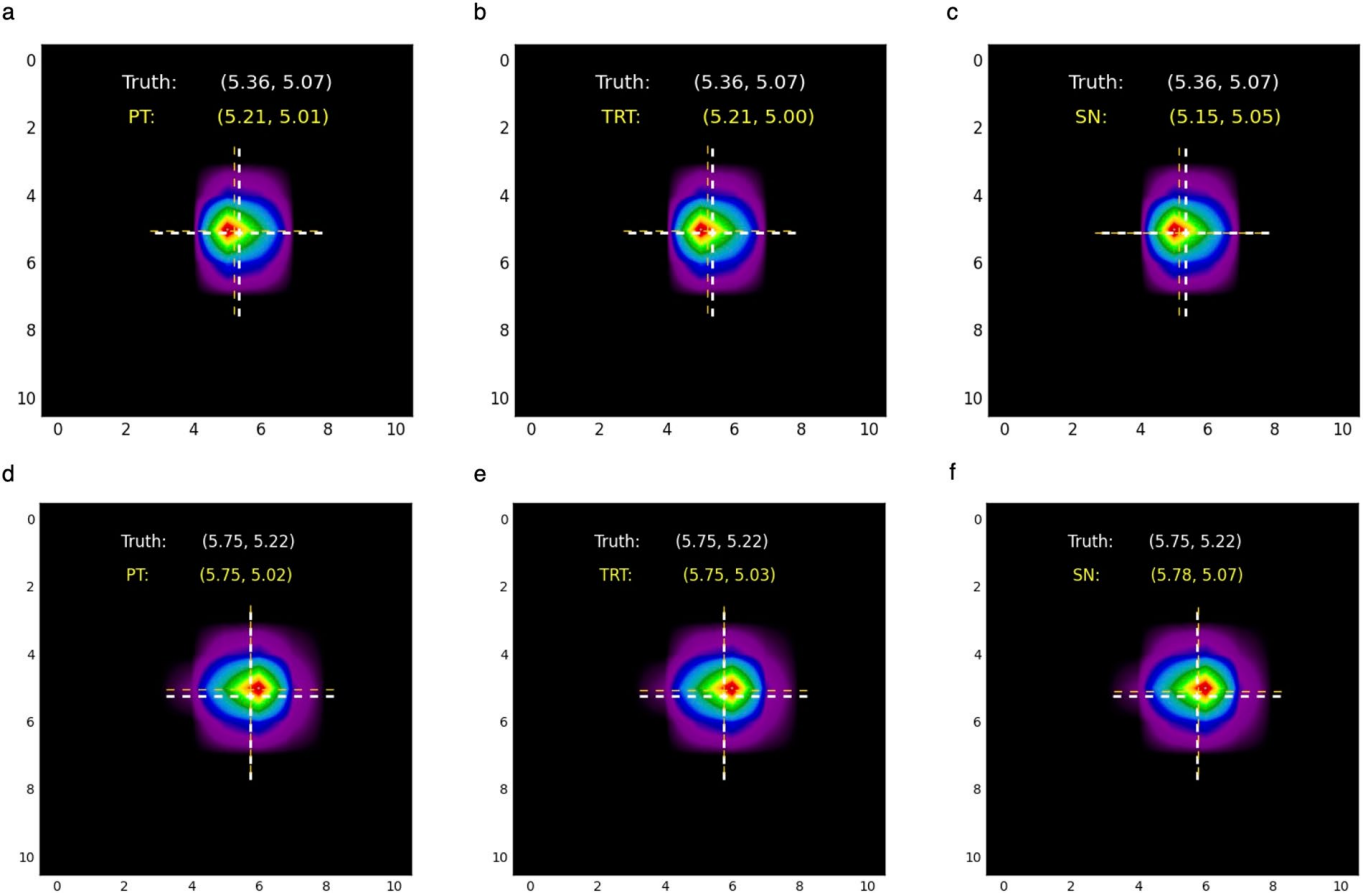
Bragg peak reconstruction. Top panels. Inference results for the identification of Bragg peak locations in an undeformed bi-crystal gold sample. From left to right, we show results for three AI models, namely: **(a)** PyTorch(PT) baseline model; **(b)** an inference optimized TensorRT(TRT) model; and **(c)** a model trained with the SambaNova DataScale^®^ (SN) system. In the panels, Truth stands for the ground truth location of Bragg peaks; PT,TRT and SN represent the predictions of our baseline PyTorch,TensorRT and SambaNova models, respectively. We produced these results by directly running these models in the ThetaGPU supercomputer, and found that 95% of the predicted peak locations in the test set are within a Euclidean distance of 0.688 *pixels* from the actual peak locations. **FAIR AI Approach. Bottom panels**. AI inference results obtained by combining DLHub, funcX and the ThetaGPU supercomputer. From left to right, we show results for our three AI models: (**d**) PT; **(e)** TRT; and **(f)** SN, which are hosted at DLHub. funcX manages the entire workflow by invoking AI models, launching workers in ThetaGPU and doing AI inference on a test set. This workflow also includes post-processing scripts to quantify the L2 norm that provides a measure for the reliability of our AI-driven regression analysis.

We used these three models to conduct AI inference in the ThetaGPU supercomputer using the validation dataset described above, i.e., 13799 Bragg peaks. We quantified the consistency of their predictions using Euclidean distance between the predicted peak locations and ground truth peak locations. We found that for all three models, 95% of the predicted peak locations in our test set are within a Euclidean distance of 0.688 pixels from the actual peak locations. In addition, the average Euclidean error is only ≈0.21 pixels, and the standard deviation of the Euclidean error is ≈0.22 pixels. For reference, the images used for this study are 11 × 11 pixels, and each pixel is 200 *μm* in size. Therefore, these results show that our three different models produce accurate and consistent predictions, even if they are trained using different optimization schemes or hardware architectures. We show a sample of these results in the top three panels of Fig. 2.

#### AI models in DLHub

AI models published in DLHub are containerized by using Docker, and include instructions for running the models with a sample test set. The models include uncertainty quantification metrics to inform users about their expected performance and realm of applicability. All trained AI models are assigned a DOI, and include descriptive metadata including the title, authors, free text description, and more (following the DataCite metadata standard), input type and shape (e.g.^11^, image maps), output type and shape (e.g.^1,2^, list of predicted Bragg peak positions) as well as examples for how to invoke each individual model. These metadata are available as JSON formatted responses through a REST API or Python SDK; or as HTML through a searchable web interface. We find that running our AI models via DLHub yields inference results that are consistent with those obtained when the models are run natively on ThetaGPU.

#### DLHub, funcX, and ThetaGPU

DLHub is configured to perform on-demand inference in Docker containers on a Kubernetes cluster hosted at the University of Chicago. The DLHub execution model leverages funcX^24^, a federated function as a service (FaaS) platform, that enables fire-and-forget remote execution. In this work, we extended that model to use a funcX endpoint deployed on ThetaGPU and configured to dynamically provision resources from ThetaGPU. ThetaGPU is an extension of the Theta supercomputer and consists of 24 NVIDIA DGX A100 nodes. Each DGX A100 node has eight NVIDIA A100 Tensor Core GPUs and two AMD Rome CPUs that provide 22 nodes with 320 GB of GPU memory and two nodes with 640 GB of GPU memory. Access to ThetaGPU is currently restricted by policy to authorized ALCF users. One approach to enabling broader access in the future could be to configure the funcX endpoint to provide access to members of a Globus Group^43^. Users could then request access to this group and, following an approval process, be granted access to run the models on ThetaGPU.

Finally, ALCF does not support Docker containers as Docker requires root privileges. As a result, we first had to transform DLHub servable containers into the Apptainer (previously Singularity) containers supported by ALCF. Apptainer provides an effective security model whereby users cannot gain additional privileges on the host system, making them suitable for deployment on high performance computing resources. After creating Apptainer containers for each AI model, we registered each with funcX along with the associated DLHub invocation function, enabling on-demand inference of the models using ThetaGPU.

#### Reproducibility of AI models

The computational framework–DLHub, funcX and ALCF–provides a ready to use, user friendly solution to harness AI models, FAIR datasets, and available computing resources to enable AI-driven discovery. We have tested the reliability of this computational framework for AI-driven discovery by processing a test set with each AI model, finding that results across models are consistent, and that these results are the same as those obtained by running AI models directly in the ThetaGPU supercomputer. The uncertainty quantification metrics we have included in our AI models also guide researchers in the use and interpretation of these AI predictions. A sample of these results is shown in the bottom panels of Fig. 2.

#### Readiness and usability of computational framework

We asked non-developers of this framework to test it and to provide feedback. They reported no issues when they followed step-by-step instructions contained in a Jupyter notebook that indicates how to load experimental data, invoke AI models from DLHUb, and then use a funcX endpoint at ThetaGPU to do inference and then compute L2 results for uncertainty quantification. With this ready-to-use, user-friendly notebook, they were able to reproduce the results we report in this article. It is worth pointing out that test users utilized their own ALCF allocation to conduct this exercise.

## Discussion

We have showcased how to FAIRify experimental datasets by harnessing the MDF and using BDBags and minids. Using these FAIR and AI-ready datasets, we described how to create and share FAIR AI models using a new set of practical definitions that we introduced in this article. Throughout this analysis, we have showcased how to harness existing data facilities, FAIR tools, modern computing environments and scientific data infrastructure to create a computational framework that is conducive for autonomous AI-driven discovery. To realize that goal, our FAIR and AI-ready datasets are published including Jupyter notebooks that provide key information regarding data type, shape and size, and how these datasets may be readily used for the creation and use of AI models. Complementing these approaches, our AI models published in DLHub include examples that illustrate how to use FAIR and AI-ready datasets for AI inference. The models also include uncertainty quantification metrics to ascertain their validity, reliability, reproducibility and statistical robustness.

**Fig. 3:**
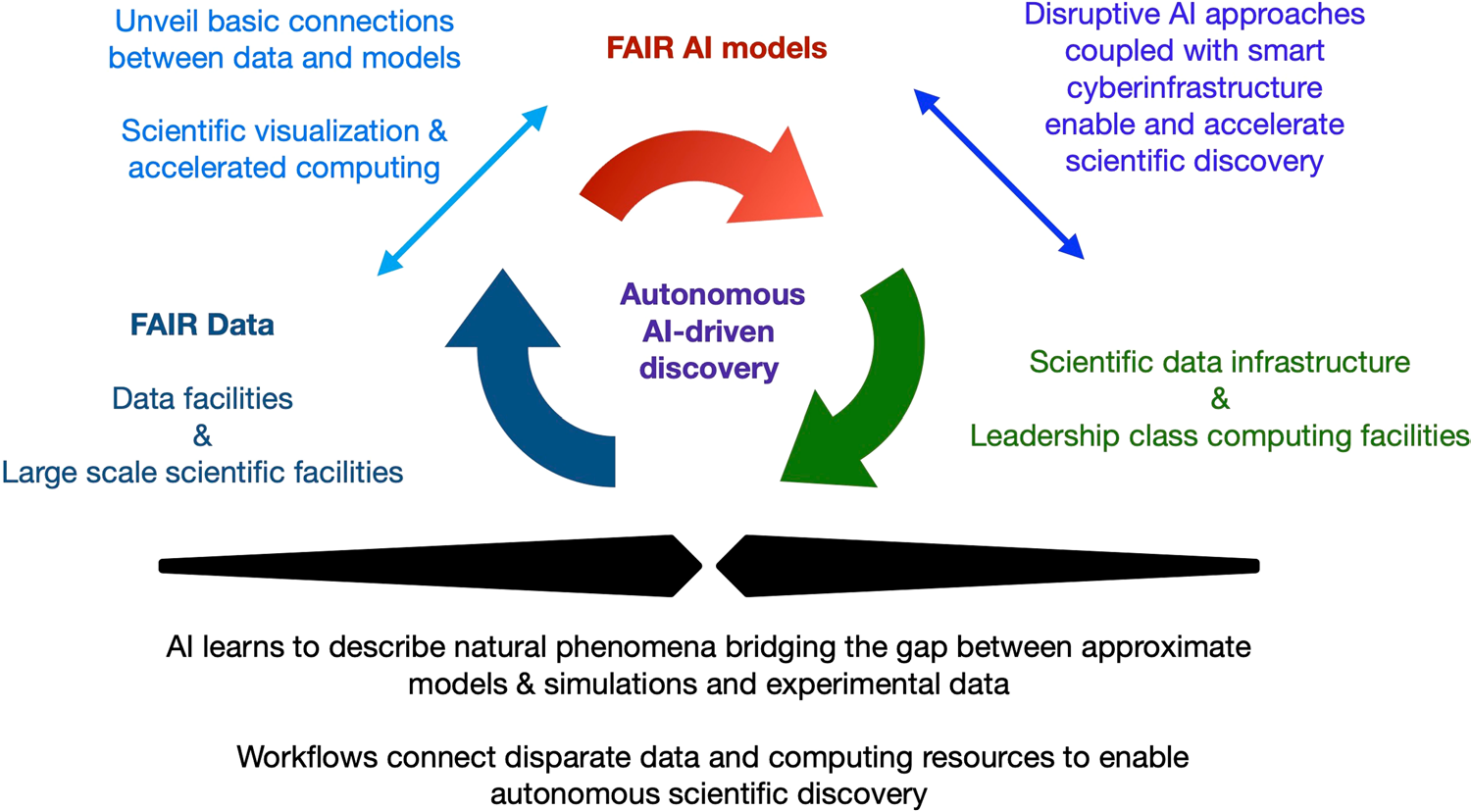
Autonomous AI-driven discovery. Vision for the integration of FAIR & AI-ready datasets with FAIR AI models and modern computing environments to enable autonomous AI-driven discovery. This approach will also catalyze the development of next-generation AI methods, and the creation of a rigorous approach that identifies foundational connections between data, models and high performance computing.

The FAIR for AI data and models approach that we describe in this article focuses on the creation of a ready-to-use, user-friendly computational framework in which data, AI, and computing are indistinguishable components of the same fabric. To realize this vision, we have brought together DLHub, MDF, funcX, and ALCF. Anticipating that this framework will be of interest to researchers and AI practitioners who are eager to explore and incorporate FAIR best practices in their research programs, we release with this manuscript all scientific software, data, and AI models used in this work. We hope that this framework is harnessed and further developed by researchers to enable advances in science, engineering and industry, as illustrated in Fig. 3.

## Data Availability

Our FAIRified datasets are published at the Materials Data facility: Training_Set^27^ and Validation_Set^28^. All FAIRified BraggNN datasets are released in HDF5 format.

## Code availability

The three AI models introduced in this article, along with the Jupyter notebook and scientific software needed to reproduce our results have been released through DLHub^36–38^. Scientific software to train these models may be found in an open source GitHub repository^33^.
